# Urinary biomarkers for early detection of platinum based drugs induced nephrotoxicity

**DOI:** 10.1186/s12882-018-1022-2

**Published:** 2018-09-04

**Authors:** Mostafa Abdelsalam, Ekramy Elmorsy, Hassan Abdelwahab, Osman Algohary, Mahmoud Naguib, Ahmed Abd El Wahab, Ahmed Eldeeb, Ehab Eltoraby, Adel Abdelsalam, Alaa Sabry, Mohamed El-Metwally, Mohamed Akl, Nahla Anber, Maysaa El Sayed Zaki, Fahad Almutairi, Tamer Mansour

**Affiliations:** 1 Mansoura Nephrology and dialysis Unit, Internal Medicine Department, Mansoura Faculty of Medicine, Mansoura University, Mansoura, Egypt; 2 Department of Forensic Medicine and Clinical Toxicology, Mansoura faculty of Medicine, Mansoura, Egypt; 3 Internal Medicine Departments, Mansoura Faculty of Medicine, Mansoura University, Mansoura, Egypt; 4 Clinical Oncology and Nuclear Medicine Department, Faculty of Medicine, Mansoura University, Mansoura, Egypt; 5 Fellow of Biochemistry, Emergency Hospital, Mansoura University, Mansoura, Egypt; 6 Clinical Pathology Department, Mansoura faculty of Medicine, Mansoura, Egypt; 7 Department of Biochemistry, Faculty of Science, University of Tabuk, Tabuk, Kingdom of Saudi Arabia; 8 Department of Population Health and Reproduction, University of California, Davis, California, USA

**Keywords:** Platinum based drugs, Cisplatin, Oxaplatin, Urinary biomarkers, Nephrotoxicity

## Abstract

**Background:**

Nephrotoxicity is a major hazard complicating the use of platinum based drugs (PBD), which can hinder using higher doses protocols to maximize the therapeutic gain. Shortage of serum creatinine level as an accurate biomarker for acute kidney injuries (AKI) necessitates searching for novel biomarkers with better sensitivity and specificity in patients on PBD.

**Methods:**

In a prospective cohort design, 132 patients receiving PBD were selected for the study. AKI was diagnosed by continuous follow up of serum creatinine level according to Kidney Disease: Improving Global Outcomes (KDIGO) guidelines 2012. Serum creatinine and urinary biomarkers (KIM-1, NGAL and cystatin C) was measured in the day of treatment and for 3 days after PBD cycle.

**Results:**

AKI occurred in 35 patients (26.52% of patients). KIM-1, Cystatin C, and NGAL showed significant increase in samples collected in the day of AKI in comparison to their corresponding basal levels (*P* <  0.0001). In addition, significant increase in urinary levels of the biomarkers in samples collected 1 day before AKI in comparison to their basal levels (*P* <  0.0001, *P* <  0.0001, and *P* = 0.013 for KIM-1, NGAL and Cystatin C respectively). Furthermore KIM-1 data showed a significant increase 2 days before serum creatinine rise in comparison to the corresponding KIM-1 levels in patients who developed AKI (*P* = 0.001).

**Conclusions:**

Urinary KIM-1, Cystatin C and NGAL can predict PBD induced AKI in earlier stages than serum createnine. KIM-1 is the most sensitive biomarker for early detection of AKI in patients receiving PBD.

## Background

Kidney is a major target for the toxicities of different chemicals and drugs. Chemicals induced nephrotoxicity was reported in about 8–60% of hospital-acquired acute kidney injuries (AKI) cases [1]. However it is not clear in these studies whether AKI was the primary reason for hospitalization or it was hospital acquired. Studies showed a marked increase in AKI incidence from 1980 to 2005 from 18 cases pxer 100,000 populations in 1980 to 365 cases per 100,000 populations [3, 4]. However, this increase in uncertain to be due to actual increase in number of cases, more ageing or increased awareness.

Diagnosis and classifications of AKI mainly depends on serum creatinine which can lead to wrong interpretation in patients whose creatinine kinetics and volume of distribution are variable or extreme [2]. On the other hand, minimal increases in serum creatinine were associated with considerable high rates of mortality which decreases the value of serum creatinine as a diagnostic and follow up tool in cases with AKI [5]. Several biomarkers were studied to detect earlier stage of renal dysfunction before it disrupts renal filtration capacity. These biomarkers are able to give accurate screening of the renal functions compared to routine renal investigations. Promising renal biomarkers include N-acetyl-β-glucosaminidase (NAG), β2-microglobulin (β2M), Cystatin-C (Cys-C), Kidney injury molecule-1 (KIM-1), Clusterin and Human neutrophil gelatinase-associated lipocalin (NGAL) [6, 7].

Strong predictive effect of urinary KIM-1 was documented previously in cases of acute tubular necrosis [8], scrub typhus [9]. In addition, KIM-1 was reported to predict proximal tubular injury after renal transplantation [10]. Regarding specificity and sensitivity of KIM-1 in cases of AKI meta-analysis study showed The estimated sensitivity of urinary KIM-1 for the diagnosis of AKI was 74.0% (95% CI, 61.0–84.0%), and specificity was 86.0% (95% CI, 74.0–93.0%) in 2979 cases from 11 published studies [11]. Urinary Cys-C had been reported to increase in tubular dysfunction [12]. Moreover, elevated urinary Cys-c was reported as bad prognostic sign in cases with non-oliguric AKI [13]. Regarding NGAL, animal models showed that it is one of the most up-regulated genes in cases of AKI [14]. Subsequent human studies confirmed its significance as a biomarker for AKI [15, 16].

Platinum based drugs (PBD), which includes Cisplatin, Oxaplatin and Carboplatin, is widely used in treatment of different malignant diseases [17]. Kidney injuries were reported in about 25–34% of patients receiving a single dose of Cisplatin [18] with increased incidence and severity of accumulative nephrotoxicity with the subsequent cycles [19, 20]. Kidney injury is considered the most dangerous side effect and can reduce the doses of Cisplatin in its designed protocols, limiting its clinical use and efficacy [21]. Some cases were reported with Oxaliplatin based chemotherapy as well [22–24].

Carboplatin is used as a safer PBD with less reported nephrotoxicity; however its myeloablative doses of 800 mg/m^2^ were shown to be nephrotoxic [25]. Also, acute interstitial nephritis was reported in 2 cases receiving Carboplatin [26]. In addition, Carboplatin induced hematuria and AKI in an old patient on Carboplatin for ovarian carcinoma was reported [27]. Furthermore, grades 1 and 2 nephrotoxicity were reported in 4–5% of cases receiving Carboplatin [28].

The aim of the current work is to assess the levels of urinary KIM-1, uNGAL and Cys-C as biomarkers for early detection of acute kidney injury in patients receiving PBD in a prospective unicenter study in Mansoura university hospitals, Egypt.

## Methods

### Ethical issues

The current study was approved by the ethical committee of Mansoura Faculty Medicine. A free voluntary informed consent was taken from all participants. Confidentiality and privacy were considered regarding participants’ personal, clinical and laboratory data.

### Patient selection

All patients enrolled in the study had received PBD for curative treatment, within the period from April 2015 to April 2016, in department of Clinical Oncology and Nuclear Medicine in Mansoura University Hospital, Dakahlya Governorate, Egypt. Patients with normal kidneys seen by pelvi-abdominal ultrasound and normal kidney functions approved by laboratory investigations were included (serum creatinine less than1.1 mg/dl, estimated glomerular filtration rates higher than 90 ml/min by The Modification of Diet in Renal Disease MDRD equation). Patients with renal dysfunction at the beginning of therapy and those who received platinum based drugs as a palliative treatment were excluded from the study. Patients with active infection, heart failure or diabetes mellitus, thyroid disorders or suprarenal diseases were also excluded. Any patient with known history of renal disease, operations or intake of a nephrotoxic drug or contrast enhanced imaging within the previous 30 days were not enrolled in the study. In addition patient with bad general condition due to anemia, dehydration, elevated liver enzymes or cerebral metastasis were also excluded. After application of exclusion criteria, 132 patients (74 males and 58 females) were selected to continue through the study. All participants were properly hydrated following hydration protocol shown in Table 1.

**Table 1:** Hydration protocol used for cases enrolled in the study during platinum based drugs (PBD)

• Before PBD:	
i. 1000 mL Sodium Chloride 0.9%	
ii. 500 ml sodium chloride with 20mmolKCL	
iii. 500 ml sodium chloride with 10 mmol MgSO4	
• During administration of PBD: PBD is given in 1000 mL Sodium Chloride 0.9% infused over 2 h	
• After PBD: 1000 mL Sodium Chloride 0.9% with 20 mmol KCL and 10 mmol MgSO4 IV	
• Monitoring during chemotherapy:	
i. Monitor urine output at regular intervals throughout chemotherapy	
ii. At the end of IV fluids, weigh the patient and review fluid input and output	
iii. Re-weigh the patient – if they have gained > 2 kg, they should be prescribed furosemide 20 mg orally and urine output monitored for a further 30 min.	
• Pre-discharge:	
i. Ensure the patient is able to maintain good oral fluid intake	
ii. Patient should be advised to drink a lot of fluids	
iii. Advise patient to contact the hospital if unable to drink due to vomiting	

### Study design

From all participants, 5 ml venous blood samples were withdrawn in the morning of PBD (just before administration of PBD) and for the subsequent 3 days of each treatment cycle. Blood samples were allowed to clot for 15 min then centrifuged for 10 min at 5000 g to obtain the serum. Creatinine was measured by using modified rate Jaffe method, using commercially available kits supplied for Human samples (Germany) [29].

For urinary markers assay, 10 ml of mid-flow urinary samples were collected in disposable urine cups without preservatives at the same time points of blood sampling. Urine samples were centrifuged at 1000 *g* for 5 min and supernatants were stored at − 20 °C. Urinary KIM-1, NGAL and Cys-C levels were measured using (ELISA kit supplied by Sunred Biological Technology, china) following the manufacturer protocol. Each sample was studied as triplicate and the average of the three readings was considered as the actual value of the urinary biomarkers. Samples with odds readings were repeated. All assays were done by the same technician using the same equipment. For biomarkers data validation inter-assay and inter-assay variability was estimated following [30].

### Statistical analysis

Data were verified. All statistical procedures were performed using PRISM 5 (GraphPad Software Inc., San Diego, CA). Repeated measures two-way ANOVA was used for statistical analysis of changes in the levels of the studied biomarkers over the course of the study. Dunnett’s multiple comparisons test was used to compare baseline values with individual data groups. ROC curve statistics were used to estimate specificity and sensitivity of the markers. Independent t-test was used to compare 2 unpaired groups of data, while Chi-square was used to compare nominal data. Statistic results were considered statistically significant when *p* values were less than 0.05.

## Results

In this study, 35 (26.5%) cases had developed AKI with increased serum creatinine during the course of the study according to KDIGO criteria (2012) [31]. There was no significant difference between patient groups, with or without AKI, regarding demographic characters, baseline laboratory data or types of cancers (Table 2). Number of patients who received Cisplatin, Oxaliplatin and Carboplatin are shown in Table 1. About 32% and 22% of patients receiving Cisplatin and Oxaliplatin respectively developed AKI, while only one patient developed AKI among six patients who received Carboplatin. Highest number of cases developed AKI in the 3rd cycle [11 patients (31.4%)] followed by the 2nd cycle [9 patients (25.7%)]. AKI was also diagnosed in 6 participants (17%) in the 1st cycle and in 4 cases after the 4th and 5th cycles.

**Table 2:** Demographic characters, types of cancers, platinum based chemotherapeutic courses and the basal laboratory data of the studied population

Parameters	AKI (+) 35 (100%)	AKI (−) 97(100%)	*p*-value	Totals 132 (100%)
Demographic data				
a. Age (Mean ± SD)	50.57 ± 13.33	49.25 ± 13.29	0.615	
1. <30Y	4 (11.8%)	7 (7.1%)		11(8.3%)
2. 30–50	10 (28.6%)	38 (39.1%)	0.68	48 (36.4%)
3. > 50	21 (60.1%)	52 (53.6%)		73 (55.3%)
b. Gender				
1. Males	17 (48.6)	57 (58.8%)	0.298	74 (56%)
2. Females	18 (51.4)	40 (41.2%)		58 (44%)
c. BMI	23.3 ± 4.1	24.3 ± 3.4	0.161	23.5 ± 4.8
Type of cancer				
a. Gastrointestinal	19 (54.3%)	61 (62.9%)		80 (60.6%)
b. Respiratory	6 (17.2)	21 (21.7%)		27 (20.5%)
c. Reproductive	2 (5.7%)	6 (6.3%)	0.5	8 (6%)
d. Neuroendocrine	2 (5.7%)	2 (2.1%)		4 (3%)
e. Osteosarcoma	2 (5.7%)	2 (2.1%)		4 (3%)
f. Others	4 (11.4%)	5 (5.25%)		9 (6.8%)
Received PBD agent:				
a. Cisplatin (50 mg/m^2^)	22 (62.9%)	49 (50.5%)		71 (53.8%)
b. Oxaliplatin (85 mg/m^2^)	12 (34.3%)	41 (42.3%)	0.45	53 (40.2%)
c. Carboplatin*	1 (2.9%)	4 (4.1%)		5 (3.8%)
d. Cispaltin (50 mg/m^2^) + Carboplatin	0	3 (3.1)		3 (2.3%)
Basal line laboratory data before PBD:				
a. Ser. Cr. (mg/dl)	0.73 ± 0.2	0.74 ± 0.3	0.85	0.73 ± 0.4
b. BUN (mmol/L)	4.5 ± 1.7	4.6 ± 1.5	0.74	4.5 ± 2
c. eGFR (ml/min)	102 ± 4	103 ± 5.2	0.3	102 ± 5.7
d. Sodium (mmol/L)	137.3 ± 5.1	136.6 ± 4.6	0.45	136.9 ± 5.8
e. Potassium (mmol/L)	3.6 ± 0.7	3.7 ± 0.6	0.42	3.6 ± 0.9
f. Calcium (mg/dl)	9.66 ± 1.05	9.50 ± 0.50	0.412	9.5 ± 1.1
KIM-1				
(0 day)	1.04 ± 0.2 (0.84–1.24)	1.2 ± 0.21 (0.99-1.41)	0.39	

BUN; Blood urea Nitrogen, Estimated glomerular filtration.* Carboplatin dose was calculated by Calvert formula

For validation of the urinary biomarkers data inert-assay and intra-assay variability of the studied biomarkers were estimated (Table 3). All the studied urinary markers (urinary KIM-1, Cys-C, and NGAL) showed significant increase in AKI group in the day of diagnosis and one day before serum creatinine increase (Tables 4 and 5). Only KIM-1 showed significant increase 2 days before serum creatinine based AKI diagnosis *P* = 0.001). KIM-1 showed the highest percentage of increase all over the course of AKI in comparison to the other studied urinary biomarkers (Table 6). Interestingly, although cisplatin was reported to be the most nephrotoxic PBD agent, there was no significant difference between the studied AKI cases regarding the level of increase of each biomarker among the patients groups following different protocols using the different PBD agents.

**Table 3:** Inter-assays and inter-assay variability of the studied urinary biomarkers ELISA assays

Urinary biomarker	Inter-assay variability	Intra-assay variability
KIM-1	High (10 ng/ml) = 8.2%	4.53%
	Low (0.1 ng/ml) =11.46%	
NGAL	High (1000 ng/ml) =9.1%	5.45%
	Low (10 ng/ml) =5.5%	
Cys-C	High (100 ng/ml) = 5.97%	6.76%
	Low (10 ng/ml) =7%

**Table 4:** Urinary biomarkers data of the studied population represented as means± standard deviation (lower limit- upper limit)

Biomarkers (ng/ml)	AKI (+) 35 cases	AKI (−) 97 cases	*p*-value
1. KIM-1			
Day 0	1.04 ± 0.2 (0.84–1.24)	1.2 ± 0.21 (0.99–1.41)	0.39
Day 1	1.5 ± 0.2 (1.3–1.7)	1.3 ± 0.2 (1.1–1.5)	0.62
Day 2	2.1 ± 0.19 (1.91–2.29)	1.2 ± 0.27 (0.93–1.47)	0.0092**
Day 3	2.25 ± 0.18 (2.07–2.43)	1.4 ± 0.2 (1.2–1.6)	0.0054**
2. NGAL			
Day 0	416 ± 46.7 (369.3–462.7)	413 ± 49.6 (363.4–462.6)	0.92
Day 1	426.3 ± 52.34 (373.69–478.64)	419.2 ± 58.4 (360.8–477.6)	0.92
Day 2	573.2 ± 39.24 (533.69–612.44)	422.8 ± 38.3 (384.5–461.1)	0.0032**
Day 3	611.6 ± 46.35 (565.25–657.95)	420.8 ± 42.3 (478.5–463.1)	0.0088**
3. Cys-c			
Day 0	14.5 ± 1.49 (13.01–15.99)	12.48 ± 1.42 (11.06–13.9)	0.22
Day 1	15.6 ± 1.7 (13.9–17.3)	12.9 ± 1.1 (11.8–14)	0.12
Day 2	18.6 ± 1.9 (16.7–20.5)	13.26 ± 0.68 (12.58–13.94)	0.013*
Day 3	22.1 ± 2.28 (19.82–24.38)	13.44 ± 0.53 (12.9–13.97)	0.003**

Day 0 means the day of PBD intake. Day 1, day2 and day3 means one, two and three days after PBD intake

* means *p*< .05, ** means *p*< .01

**Table 5:** Urinary level of KIM-1, NGAL and Cys-C among the studied population with (AKI+) or without acute Kidney injury (AKI-)

Biomarkers (ng/ml)	AKI (+) 35 cases		AKI (−) 97 cases	
	Dunnett’s multiple comparisons	Two way ANOVA *p*-value		Two way ANOVA *p*-value
1. KIM-1				
Day 0 versus day 1	< 0.048*		0.58	0.94
Day 0 versus day 2	< 0.0026**	< 0.0002***	1	
Day 0 versus day 3	< 0.0015**		0.29	
Day 1 versus day 2	0.019*		0.63	
Day 2 versus day 3	0.37		0.36	
2. NGAL				
Day 0 versus day 1	0.81		0.89	
Day 0 versus day 2	0.011*	0.0015**	0.81	0.99
Day 0 versus day 3	0.006**		0.86	
Day 1 versus day 2	0.017*		0.95	
Day 2 versus day 3	0.33		0.94	
3. Cys-c				
Day 0 versus day 1	0.445		0.71	0.69
Day 0 versus day 2	0.045*	0.0045**	0.45	
Day 0 versus day 3	0.007**		0.35	
Day 1 versus day 2	0.11		0.66	
Day 2 versus day 3	0.11		0.87	

Day 0 means the day of PBD intake. Day 1, day2 and day3 means one, two and three days after PBD intake. Two way ANOVA *p*-values were shown for each biomarker. Intergroup differences significance was calculated by Dunnett’s multiple comparisons test. * means *p*-vlaue< 0.05, ** means *p*-value< 0.01, while *** means *p*-value < 0.001

**Table 6:** Percentages of increase in the urinary biomarkers KIM-1, NGAL and Cys-C over the course of the study

Parameters	KIM-1	NGAL	Cys-C
	M% ± SD	M% ± SD	M% ± SD
Basal levels versus levels 2 days before AKI	44.23 ± 19.2	2.5 ± 3	7.31 ± 11.7
Basal levels versus levels 1 days before AKI	101.9 ± 18.27	37.8 ± 2.8	27.98 ± 13.1
Basal levels versus levels in the days AKI diagnosis	116.3 ± 17.31	47.02 ± 2.72	52.2 ± 15.7

Percentage of increase were calculated in each patient with individually. Data is represented as means ±standard deviation of all patients biomarkers increase percentages

**Fig. 1:**
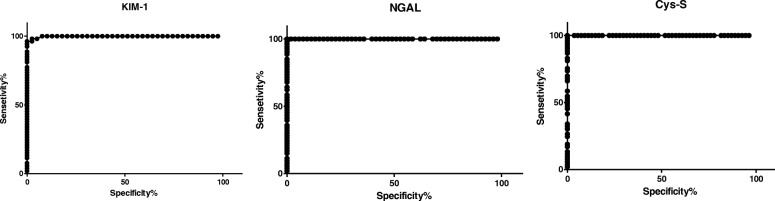
Sensitivity and specificity of the different urinary biomarkers in cases of AKI

The basal levels of KIM-1, Cys-C, and NGAL (before the chemotherapy) were compared with their levels after treatment in the AKI group. ROC curve shows levels of cut off with the highest sensitivity and 100% specificity (Table 7 and Fig. 1).

**Table 7:** Comparison of the levels of the urinary markers in patients with AKI (one before AKI diagnosis) and their corresponding level in patients without AKI

Biomarkers	AUC (95% Confidence Interval)	*p*-value	Cut-off	Sensitivity%	Specificity%
KIM-1	0.9983 (0.9946 to 1.002)	< 0.0001	< 1.685	96.23	100.0
			< 1.730	96.23	97.44
			< 1.750	98.11	97.44
NGAL	1 (1–1)	< 0.0001	< 498.0	100.0	100.0
			< 508.2	100.0	98.11
			< 511.7	100.0	96.23
Cys-S	1 (1–1)	< 0.0001	< 15.43	100.0	100.0
			< 16.65	100.0	96.30
			< 16.75	100.0	94.44

## Discussion

PBD is an efficient chemotherapy used in different malignancies; however it carries high risk of AKI even in cases with good hydration. In the current study, 3 well-known urinary biomarkers (KIM-1, Cys-C, NGAL) of kidney injury were evaluated for early diagnosis of PBD-induced AKI in comparison to the routinely used serum creatinine. In the last years, several studies tried to use KIM-1, Cys-C and NGAL as sensitive biomarkers for early detection of AKI among patients receiving PBD. To the best of our knowledge, this study is the first study which tried to combine those three urinary biomarkers in a one comparative study and with the new design for AKI detection.

In the current study of 132 patients who received PBD, 26.5% developed AKI. In recent studies, the incidence of AKI among patients on Cisplatin based chemotherapy was about 34% [32, 33]. This difference may be due to the higher Cisplatin doses received in these studies (≥ 60 mg/m^2^). The current data revealed 22% as incidence of AKI among patient received Oxaliplatin, which is relatively higher than expected. This higher incidence may be related to the followed protocol dose, the general condition of the patient and their compliance with the followed hydration protocols. Some of our patients received Carboplatin and Oxaplatin as an alternative chemotherapeutic drugs in their protocol of therapy with less nephrotoxic effect.

In the current study basal levels of urinary biomarkers were used and compared with the subsequent value in the same individual using repeated measures statistics as it will be more accurate than the comparisons of means as the basal levels of these biomarkers vary among the healthy population.

AKI was diagnosed in the 3rd day of PBD administration using serum creatinine based on KDIGO 2012 criteria [31]. The current data revealed that all the studied markers showed significant increase in samples collected one day before AKI diagnosis (*P* = 0.004, *P* = 0.009, and *P* = 0.01 for KIM-1, Cys-C and NGAL respectively). Even more, KIM-1 data showed significant increase 2 days before serum creatinine rise. Interestingly, the increase of the tested markers was specific to renal injury and did not happen in patients without renal drawbacks of PBD.

These changes are in agreement with several previous studies. Gaspari et al. (2010) [34] concluded that the increase of NGAL at day 2 post treatment was a significant predictor of subsequent AKI. The validity of both NGAL and Cys-C to screen Cisplatin-AKI was evaluated by Lin et al. (2013) [35]. Lin and his collages demonstrated that NGAL showed significant increase 12 h after Cisplatin in cases that developed AKI. In the same study, urinary Cys-C was a poor marker for AKI induced by Cisplatin. These different results by Lin et al. may be due to small number of patients (33 patients with only 10 cases showed AKI), different AKI definition (according to RIFLE criteria), and different methods and time points for urinary cystatin measurements, which differ from our study design. In addition Cys-C in their study was expressed as a ratio to urinary creatinine.

Moreover, Tekce et al. (2015) [36] studied 22 patients in Turkey and showed that KIM-1 was significantly increased in 8 cases that developed AKI. Urinary KIM-1 levels were significantly increased 24 h after Cisplatin in cases diagnosed in the 3rd day after treatment, which is in agreement with the current data. Data regarding the second day after Cisplatin was not collected in their paper.

Regarding the changes of the biomarkers in cases of PBD induced AKI during the course of the study, there is discordance between our findings and Lin et al., study who showed early increase in the biomarkers from the basal line within 6 h in cases developed AKI, then the levels declined (but still above the basal levels) to be increased again for another peak within 48 h. This difference in the data may be due to the different hydration protocol and when the samples are collected in relation to the fluids intake as hydration can dilute the urinary biomarkers. In addition they have taken more frequent samples every hour during the whole course of the study.

Interestingly, NGAL showed the highest significant increase in the day of serum creatinine based AKI diagnosis in comparison to their corresponding basal levels. These finding have high clinical significance as they show the benefit of these biomarkers for early screening of PBD induced AKI and showed that serum creatinine increase is a late event in the cascade of events of AKI progress course.

The current study data is important for early screening of cases of AKI. However it has its own limitations as other larger multicenter studies are recommended for more robustness of data and will check the data in different groups of patient with different cancers and receiving different PBD and different grades of AKI. Such studies are able to detect sharper cut-off point with higher sensitivity and specificity for the different markers specially KIM-1 which showed better results as an early biomarker.

## Conclusion

Acute kidney injury is still a challenging side effect of platinum based agents, in spite of reduced dosage protocols and proper hydration. Urinary KIM-1, Cystatin C and NGAL can predict PBD induced AKI in earlier stages than serum creatinine. KIM-1 is the most sensitive biomarker for early detection of AKI in patients receiving PBD.

## Abbreviations

AKI: Acute Kidney Injury
Cys-C: Cystatin-C
KDIGO: Kidney Disease: Improving Global Outcomes
KIM-1: Kidney injury molecule-1
NAG: N-acetyl-β-glucosaminidase
NGAL: Human neutrophil gelatinase-associated lipocalin
PBD: platinum based drugs
β2M: β2-microglobulin
